# Pigment epithelium-derived factor hinders photoreceptor cell death by reducing intracellular calcium in the degenerating retina

**DOI:** 10.1038/s41419-018-0613-y

**Published:** 2018-05-11

**Authors:** Antonella Comitato, Preeti Subramanian, Giandomenico Turchiano, Monica Montanari, S. Patricia Becerra, Valeria Marigo

**Affiliations:** 1 Department of Life Sciences, University of Modena and Reggio Emilia, Modena, Italy; 2 Section of Protein Structure and Function, Laboratory of Retinal Cell and Molecular Biology, NEI, National Institutes of Health, Bethesda, MD 20892 USA; 3 Center for Neuroscience and Neurotechnology, University of Modena and Reggio Emilia, Modena, Italy; 4 Present Address: Institute for Cell and Gene Therapy & Center for Chronic Immunodeficiency - University of Freiburg, Freiburg, Germany

## Abstract

Calcium ions play a critical role in neuronal cell death. Pigment epithelium-derived factor (PEDF) is a promising neuroprotective protein for photoreceptor cells but the mechanisms mediating its effects against retinal degeneration are still not well characterized. We addressed this question in the *rd1* degenerating mouse retina that bears a mutation in the *Pde6b* gene encoding one subunit of the phosphodiesterase enzyme. Loss of phosphodiesterase activity in rod photoreceptor cells increases cyclic guanosine monophosphate (cGMP) levels leading to a rise in intracellular calcium. Short-term treatments with recombinant human PEDF protein decreased intracellular calcium in photoreceptors in vivo. Taking advantage of calcium pump blockers, we defined that PEDF signaling acts on PMCA calcium pumps to lower intracellular calcium. PEDF restrained cell death pathways activated by high calcium levels and engaging calpains, BAX and AIF. The neurotrophic effects were mediated by the PEDF receptor (PEDF-R), encoded by the *PNPLA2* gene. Finally, peptides containing the neurotrophic domain of PEDF targeted these same cell death pathways in vivo. The findings reveal rescue from death of degenerating photoreceptor cells by a PEDF-mediated preservation of intracellular calcium homeostasis.

## Introduction

Retinal degeneration is an inherited disease linked to mutations in >100 genes and this genetic heterogeneity hampers the development of a cure. Although gene therapy was developed for specific forms of the disease, unfortunately, only a limited number of patients can benefit from such an exquisite type of therapy. In recent years, we and others have reported several lines of evidence for common molecular mechanisms that are activated during photoreceptor cell death in different models of the disease^1,2^. The application of neurotrophic factors to target common cell death mechanisms is an attractive strategy for treating more than only one form of this group of diseases. Neuroprotective activities of several molecules were reported in different models of retinal degeneration and in clinical trials^3–14^. However, the use of neuroprotective factors requires deep knowledge on the molecular mechanism underlying their effects to better interpret the outcomes of the treatment.

Pigment epithelium-derived factor (PEDF) is a protein implicated in the survival and normal function of photoreceptor cells^15^. PEDF is found in the healthy human eye and its levels are altered in eyes affected by retinal degenerative processes^16–20^. In human and murine eyes with retinal degeneration, PEDF levels are reduced and in animal models of retinopathies PEDF treatments protect the neuroretina, attenuate angiogenesis and neovessel invasion, and prevent loss of visual function^15,16,18,20,21^. In the retina, PEDF is preferentially secreted from the apical-lateral side of the retinal pigment epithelium (RPE) toward the photoreceptors, where it acts on photoreceptor morphogenesis, neurite outgrowth and survival^22,23^. PEDF also promotes retinal stem cell expansion in vitro^24^. PEDF is a secreted glycoprotein bearing separated functional domains for neurotrophic and antiangiogenic effects^25–28^. Photoreceptors and ganglion cells in the retina express receptors for PEDF^29^ and one of these is PEDF receptor (PEDF-R) encoded by the patatin-like phospholipase domain-containing 2 (*PNPLA2*) gene^30^. PEDF-R is a phospholipase membrane protein expressed at the surface of retinal cells and is localized at the inner segment of the photoreceptors and, at lower levels, also in other retinal cell types^30^. PEDF-R mediates retinal cell survival activity of PEDF in vitro and in vivo^11,31^.

Intravitreal injections of recombinant human PEDF protein significantly decreased photoreceptor cell death in rodent models of retinal degeneration caused by genetic mutations or by light damage^11,13,14,32,33^. Ectopic expression of PEDF in the Royal College of Surgeons (RCS) rats by subretinal delivery using a lentiviral system showed that PEDF reduces nuclear translocation of apoptosis-inducing factor (AIF), as well as increases B-cell lymphoma 2 (BCL2) protein levels in photoreceptors^12^. We recently showed that peptides derived from the domain of human PEDF protein that confer the functions associated with neuronal differentiation and survival (residues 98–114) are strongly neuroprotective when injected into the eye of a murine model of retinal degeneration, the retinal degeneration 1 (*rd1*) mouse, and act through PEDF-R^11^. Studies published so far indicated that PEDF is a promising neuroprotective protein for retinal degeneration but the mechanisms mediating PEDF effects in the retina are still not well characterized. This lack of information hampers the molecular and pharmacological evaluation of the beneficial effects of PEDF as a prospective therapeutic agent for the retina.

To better characterize targets of the protective activity of PEDF in photoreceptors, we analyzed cell death pathways in *rd1* mutant retinas by treatment with purified recombinant PEDF protein and short PEDF peptide fragments^11^ via intravitreal injections. The *rd1* mouse model bears a mutation in the *Pde6b* gene and is associated with increased levels of cGMP due to the lack of activity of the phosphodiesterase enzyme (PDE6)^34^. cGMP, not hydrolyzed by PDE6, accumulates inside the cells activating several intracellular signals and, among them, provokes an influx of Ca^2+^ ions by binding to cGMP-gated cation (Na^+^/Ca^2+^) channels^35,36^. Calpain proteases respond to changes in intracellular Ca^2+^ and are over-activated in *rd1* mutant photoreceptors^9,37,38^. Activation of calpains triggers several downstream responses in the *rd1* mutant retina, such as activations of cathepsin D and BAX^2^. AIF, a cell death executioner, exits from mitochondria through a pore formed by BAX upon cleavage by calpains and translocates into the nucleus leading to chromatin fragmentation^39–41^. We, thus, evaluated intracellular calcium content and calpain activation and we determined the levels of BAX, BCL2 and AIF proteins after treatment with PEDF in vivo. We explored in vitro and in vivo the role of PEDF on the extrusion of calcium using specific Ca^2+^ pump inhibitors in models of the disease. Our findings lead to discussions of a novel pathway for the PEDF neurotrophic effects against retinal degeneration.

## Results

### PEDF protects the degenerating retina by decreasing intracellular calcium

**Fig. 1:**
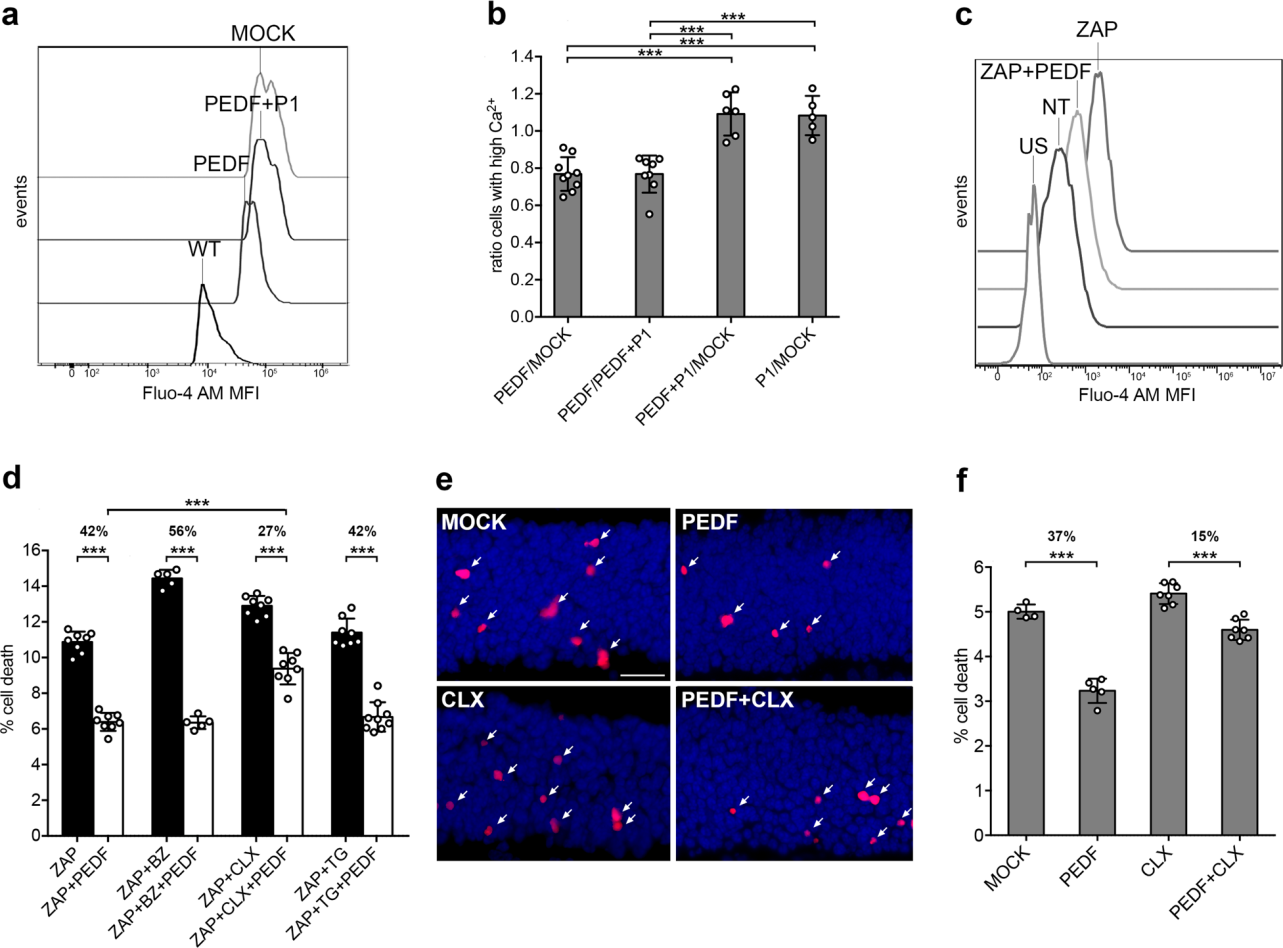
Decreased intracellular Ca2+ after treatment with PEDF. **a** Cytofluorimetric analysis of Ca^2+^, as evaluated by Fluo-4 AM labeling, in *rd1* mutant photoreceptors not treated (MOCK) or treated with 6 pmol of PEDF (PEDF) or co-treated with PEDF and 10 molar excess of P1 peptide (PEDF + P1). As control, we show data from wild-type photoreceptors (WT). The levels of calcium (medium fluorescence intensity, MFI) in cells (events) were reduced in the presence of PEDF (PEDF vs MOCK and PEDF vs PEDF + P1). **b** Ratio of photoreceptor cells with high levels of Ca^2+^ (see parameters in Supplemental figure S1), as evaluated by Fluo-4 AM labeling and cytofluorimetric analysis, in PEDF treated compared with contralateral eyes treated either with vehicle (MOCK) or with PEDF and 10 molar excess of P1 peptide (PEDF + P1). Data are shown as means ± SD (*N* = 4–9; ***P ≤ 0.001). **c** Cytofluorimetric analysis of Ca^2+^, as evaluated by Fluo-4 AM labeling, in 661W cells stressed with 500 μM zaprinast (ZAP), to block PDE6 function and to mimic the *rd1* mutation, and in 661W cells treated with Zaprinast and PEDF (ZAP + PEDF). As control, we show data from cells unstained with Fluo-4 AM (US) or not treated with zaprinast (NT). The levels of calcium (medium fluorescence intensity, MFI) in cells (events) were reduced in the presence of PEDF in cells stressed with zaprinast (ZAP + PEDF vs ZAP). **d** 661W cells were stressed with 500 μM zaprinast (ZAP) that caused cell death, as assessed by TUNEL staining. PEDF neuroprotective effects were maintained after exposure to 100 μM 3′,4′-dichlorobenzamyl (BZ) or 200 nM Thapsigargin (TG). PEDF neuroprotection was reduced when cells were exposed to 10 μM caloxin (CLX). The percentage of PEDF neuroprotection is reported above the *P*-value. Data are shown as means ± SD (*N* = 5–8; *** *P* ≤ 0.001). **e** Sections of *rd1* mutant retinas either treated with vehicle (MOCK) or with 6 pmol of PEDF (PEDF) or with 100 μM of CLX or with PEDF and CLX (PEDF + CLX). The outer nuclear layer of the retina, containing the photoreceptor nuclei (blue), is shown after TUNEL staining (red, arrows). Scale bar: 20 μm. **f** Quantification of cell death in *rd1* mutant retinas either treated with vehicle (MOCK) or with 6 pmol of PEDF (PEDF) or with 100 μM of CLX or with PEDF and CLX (PEDF + CLX). The percentage of neuroprotection is reported above the *P*-value. Data are shown as means ± SD (*N* = 4–6; ***P ≤ 0.001)

We recently defined that doses of 6 pmol per eye of recombinant PEDF significantly protect *rd1* mutant photoreceptor cells by lowering cell death by about 40%^11^. Applying this same injection paradigm, that is, intravitreal delivery in *rd1* mice at postnatal-day 11 (PN11) and analysis 16 h later at PN12, we assessed cell death pathways in the *rd1* model of retinal degeneration. First we assayed for intracellular Ca^2+^ content in the *rd1* photoreceptors after treatment with PEDF because retinal degeneration in the *rd1* model is characterized by influx of Ca^2+^ ions^35,37^. Using the Fluo-4 AM fluorescent dye, we compared PEDF-treated with contralateral mock-treated samples by cytofluorimetric analysis. We consistently found a decreased number of photoreceptors with high intracellular Ca^2+^ after treatment with PEDF (Figs. 1a, b and Supplemental figure S1 a-d). The specificity of this outcome was investigated with the use of the P1 peptide that blocks the binding of PEDF to its receptor PEDF-R^11,31^. When we co-injected PEDF with 10-fold molar excess of the P1 interfering peptide, the PEDF inhibitory effects on intracellular Ca^2+^ levels were abolished (Figs. 1a, b).

Photoreceptors outer segments, where the phototransduction cascade is initiated, possess the cGMP-gated channel (CNGC) for Ca^2+^ entrance and one Ca^2+^ clearance pathway through the NCKX (Na^+^/Ca^2+^-K^+^ exchanger)^42^. The highest intracellular Ca^2+^ reservoir in photoreceptors is the endoplasmic reticulum (ER) where Ca^2+^ uptake is mediated by the sarco-ER Ca^2+^ ATPase (SERCA). Ca^2+^ clearance from the cytosol is regulated by SERCA and by PMCA (plasma membrane Ca^2+^ ATP-ase) pumps^42^. We investigated if PEDF could modulate intracellular Ca^2+^ by acting on any of these pumps. To this aim, we modeled the loss-of-function of PDE6 using 661W cell cultures treated in vitro with zaprinast, a PDE6 inhibitor that increases intracellular cGMP. Zaprinast increased intracellular Ca^2+^ and also induced cell death in 661W cells (Figs. 1c, d and Supplemental Figures S1 e and S2 a). Paralleling our in vivo findings (Fig. 1a and Kenealey et al.^11^), the in vitro model showed that PEDF could decrease intracellular calcium, as well as photoreceptor cell death by about 40% (Figs. 1c, d and Supplemental Figure S2 a). To identify the intracellular targets of PEDF, we blocked one by one the three clearance pumps/exchangers with specific drugs. We first tested 3′,4′-dichlorobenzamyl (BZ), a blocker of NCKX pumps, and caloxin (CLX), a blocker of PMCA, and defined concentrations able to increase intracellular calcium and thus to block the pumps (Supplemental Figure S2 b). We also tested thapsigargin (TG), a blocker of SERCA, by analysis of activation of the ER stress marker PERK, which resulted phosphorylated (Supplemental Figure S2 c). It is worth noting that the three drugs at the tested concentrations did not increase 661W cell death by themselves after 16 h of treatment (data not shown) and modestly increased death in cells that had been stressed with zaprinast (Fig. 1d). Interestingly, only CLX, which blocks PMCA, significantly attenuated the PEDF-mediated neuroprotective effects suggesting that PEDF may act through these plasma membrane pumps (Fig. 1d). The block of SERCA by TG, and NCKX by BZ, did not affect PEDF neuroprotection (Fig. 1d), discarding these pumps from mediating Ca^2+^ clearance, at least in this model. We confirmed the effects of PEDF on PMCA in an alternative in vitro model, that is, rod-like cells differentiated from *rd1* mutant neurospheres^37^. PEDF protected *rd1* mutant rod-like cells from cell death and CLX interfered with the neuroprotective activity (Supplemental Figure S3). To examine the direct effect of PEDF on the regulation of intracellular Ca^2+^, we treated 661W cells with A23187, an ionophore that allows calcium influx, as also shown in retinas in vivo^43^. PEDF strongly reduced cell death in A23187-treated 661W cells and, most importantly, the PMCA blocker CLX interfered with the protective activity of PEDF (Supplemental Figure S2 d).

To confirm in vivo that PEDF decreases [Ca^2+^]_i_ by targeting PMCA, PEDF was intravitreally co-injected with CLX in *rd1* mutant eyes. We defined that 100 μM concentration of CLX was able in vivo to increase [Ca^2+^]_i_ in photoreceptors with low toxicity (Supplemental Figure S4). When PEDF was co-injected with CLX, the neuroprotective activity was significantly reduced (Figs. 1e, f).

These results indicated that PEDF acted on PMCA to reduce intracellular calcium at early steps of the rod photoreceptor cell death cascade.

### Calpain activation is restrained by PEDF

**Fig. 2:**
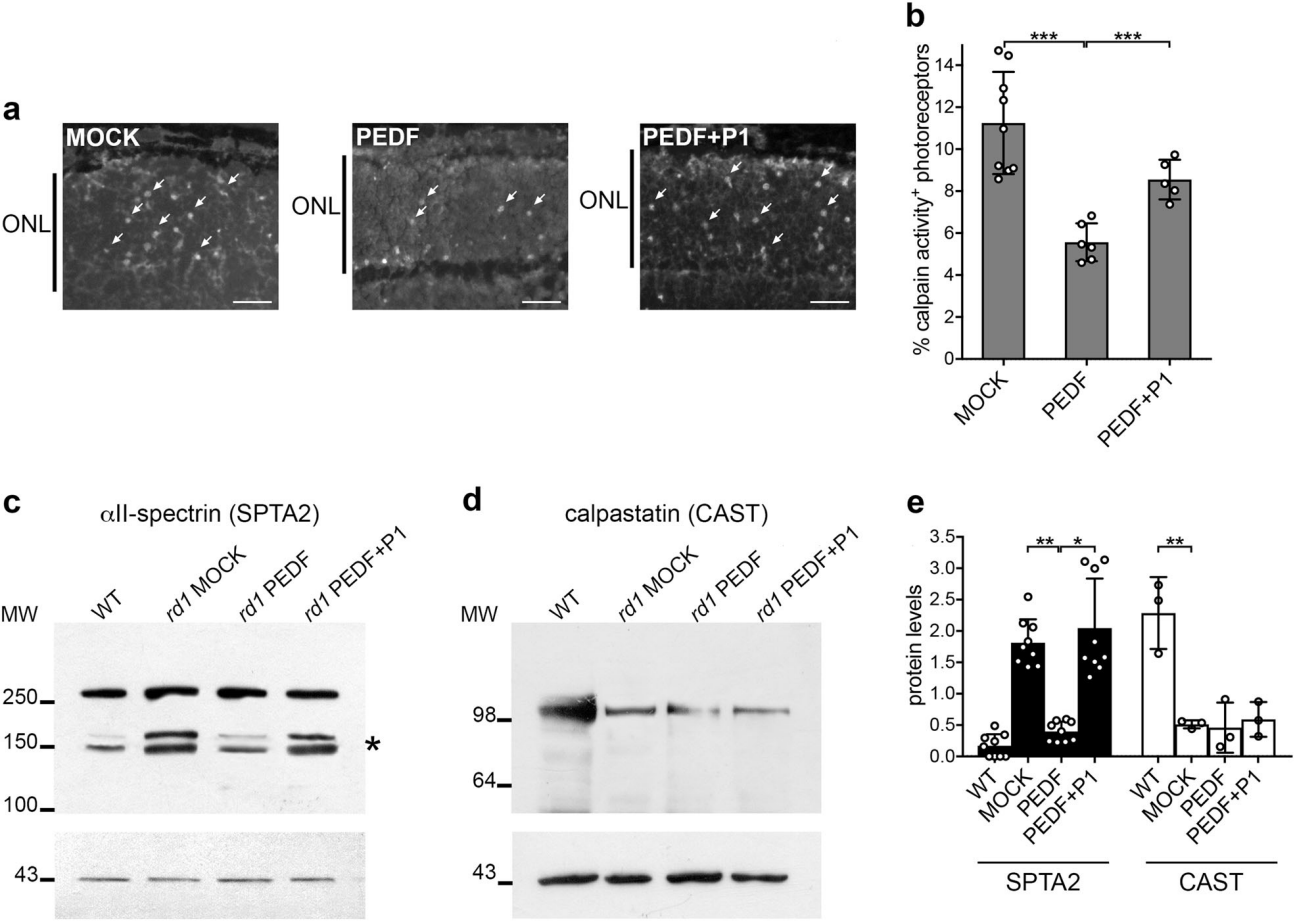
PEDF constrains activation of calpains. **a** Calpain activity, as detected by exposure of frozen unfixed *rd1* retina sections to a fluorescent calpain substrate, shows a decrease in number of cells with high calpain activity (fluorescent spots in the outer nuclear layer, arrows) in retinas treated with PEDF but not in retinas treated with PEDF and P1 blocking peptide (PEDF + P1). ONL outer nuclear layer containing photoreceptors. Scale bar: 20 μm. **b** Percentages (±SD) of photoreceptors with activated calpains counted in the ONL of retinal sections passing through the optic nerve, as shown in panel (**a**) (*N* = 5). **c**, **d** Total protein extracts from wild type (WT) and *rd1* mutant mouse retinas were analyzed by immunoblots after treatment with either vehicle (MOCK) or PEDF or PEDF and P1 blocking peptide (PEDF + P1). The analysis with an anti-αII-spectrin antibody (SPTA2, **c**) shows a decrease of the 145–150 kDa fragments resulting from calpain cleavage (asterisk) in PEDF-treated retinas. Analysis of calpastatin (CAST, **d**) shows a decrease of the protein in mutant retinas (MOCK) compared with wild-type retinas but no change after treatment with PEDF. The immunoblots were normalized using anti-actin antibodies (lower panels). MW molecular weight markers are shown in kDa. **e** Quantification by ImageJ of at least three immunoblots from biological replicates as the one shown in panel (**c**) (cleaved αII-spectrin, black bars) and panel (**d**) (white bars) (±SD; *N* = 3). ***P ≤ 0.001; **P ≤ 0.01; *P ≤ 0.05

After defining that PEDF reduces intracellular calcium, we evaluated activation of calcium-dependent proteases calpains by an in situ calpain activity assay^2,9,37,38^ and by assessing the cleavage of αII-spectrin, a substrate for calpains^44^. PEDF attenuated calpain activation in *rd1* mutant photoreceptors and co-injection with the P1 blocking peptide at 10-fold molar excess to PEDF reversed the effect (Figs. 2a, b). Reduced calpain activity after exposure to PEDF was confirmed by the decrease of the amount of the 145–150 kDa fragments of αII-spectrin derived from calpain cleavage in *rd1* mutant retinas (Fig. 2c, asterisk, and e). Co-injection of the P1 peptide blocked the effects of PEDF on calpain activity, implying involvement of PEDF-R.

We speculated that the attenuated calpain activation was a result of increased levels of calpastatin, its endogenous inhibitor^45^. Although we observed decreased calpastatin levels in the *rd1* retina, as previously published^38^, PEDF did not restore wild-type levels of the calpain inhibitor (Figs. 2d, e and Supplemental Figures S5 a).

These results implied that the decrease of intracellular calcium levels mediated by PEDF contributed to the attenuation of activated calpains independent of calpastatin.

### PEDF limits activation of BAX in the degenerating retina

**Fig. 3:**
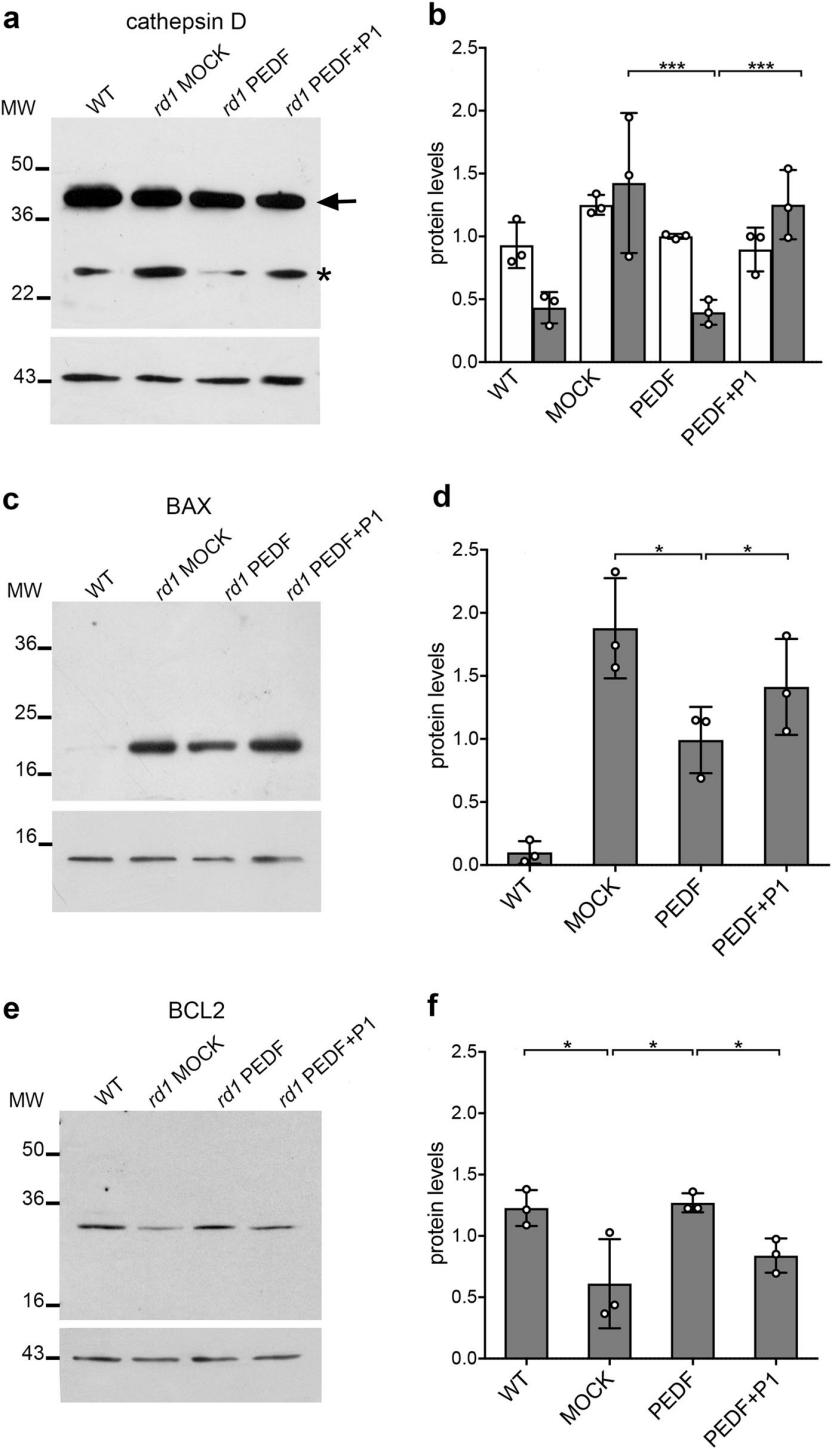
PEDF effects on Cathepsin D, BAX and BCL2. Protein analyses on wild-type (WT) and *rd1* mutant mouse retinas treated with either vehicle (MOCK) or PEDF or PEDF and P1 blocking peptide (PEDF + P1). **a** Total protein extracts were analyzed by immunoblot with an anti-cathepsin D antibody. The antibody recognizes both uncleaved cathepsin D (arrow) and cleaved/activated cathepsin D (asterisk). The immunoblot was normalized using anti-actin antibodies (lower panel). **b** Quantification by ImageJ of three immunoblots from biological replicates as the one shown in panel (**a**) (±SD; *N* = 3). White bars show values for uncleaved cathepsin D and gray bars show values for activated/cleaved cathepsin D. **c** Analysis of the BAX protein in mitochondria enriched extracts. The immunoblot was normalized with anti-cytochrome *c* antibodies (lower panel). **d** Quantification by ImageJ of three immunoblots from biological replicates as the one shown in panel (**c**) (±SD; *N* = 3). **e** Total protein extracts were analyzed by immunoblot with an anti-BCL2 antibody. The immunoblot was normalized using anti-actin antibodies (lower panel). **f** Quantification by ImageJ of three immunoblots from biological replicates as the one shown in panel (**a**) (±SD; *N* = 3). MW molecular weight markers are shown in kDa. ***P ≤ 0.001; *P ≤ 0.05

We assessed the activation of cathepsin D by immunoblotting and found that the cleaved/activated form of cathepsin D (Fig. 3a, asterisk) was significantly decreased after PEDF exposure when compared with mock-treated retinas or to retinas co-injected with 10-fold molar excess of blocking P1 peptide over PEDF (Figs. 3a, b).

Calpains and cathepsin D activate BAX, which translocates to the mitochondria in the retina and in other tissues^2,46^. We evaluated BAX translocation to the mitochondrial membrane by immunoblotting of mitochondrial-enriched protein extracts. PEDF decreased mitochondrial BAX proteins levels, which again was reversed with 10-fold molar excess of P1 peptide over PEDF (Figs. 3c, d and Supplemental Figures S5 b).

Activation of BAX can be modulated also by other BH3 family members and among those the anti-apoptotic BCL2 protein that we reported to be reduced in the *rd1* retina and in other murine models of RP relative to wild-type mice^2^. We evaluated BCL2 protein and found that PEDF restored BCL2 levels and the rescue was blocked when the P1 interfering peptide was co-injected with PEDF (Figs. 3e, f and Supplemental Figures S5 c).

These observations implied that PEDF attenuated BAX activation in photoreceptor cells and increased BCL2 levels. Moreover, interference of PEDF binding to PEDF-R by the P1 peptide reversed the PEDF effects on cathepsin D and BAX activation, as well as on BCL2 levels implying a role for the PEDF–PEDF-R interaction in the attenuation of the cell death pathway.

### PEDF impairs translocation of AIF in the degenerating retina

**Fig. 4:**
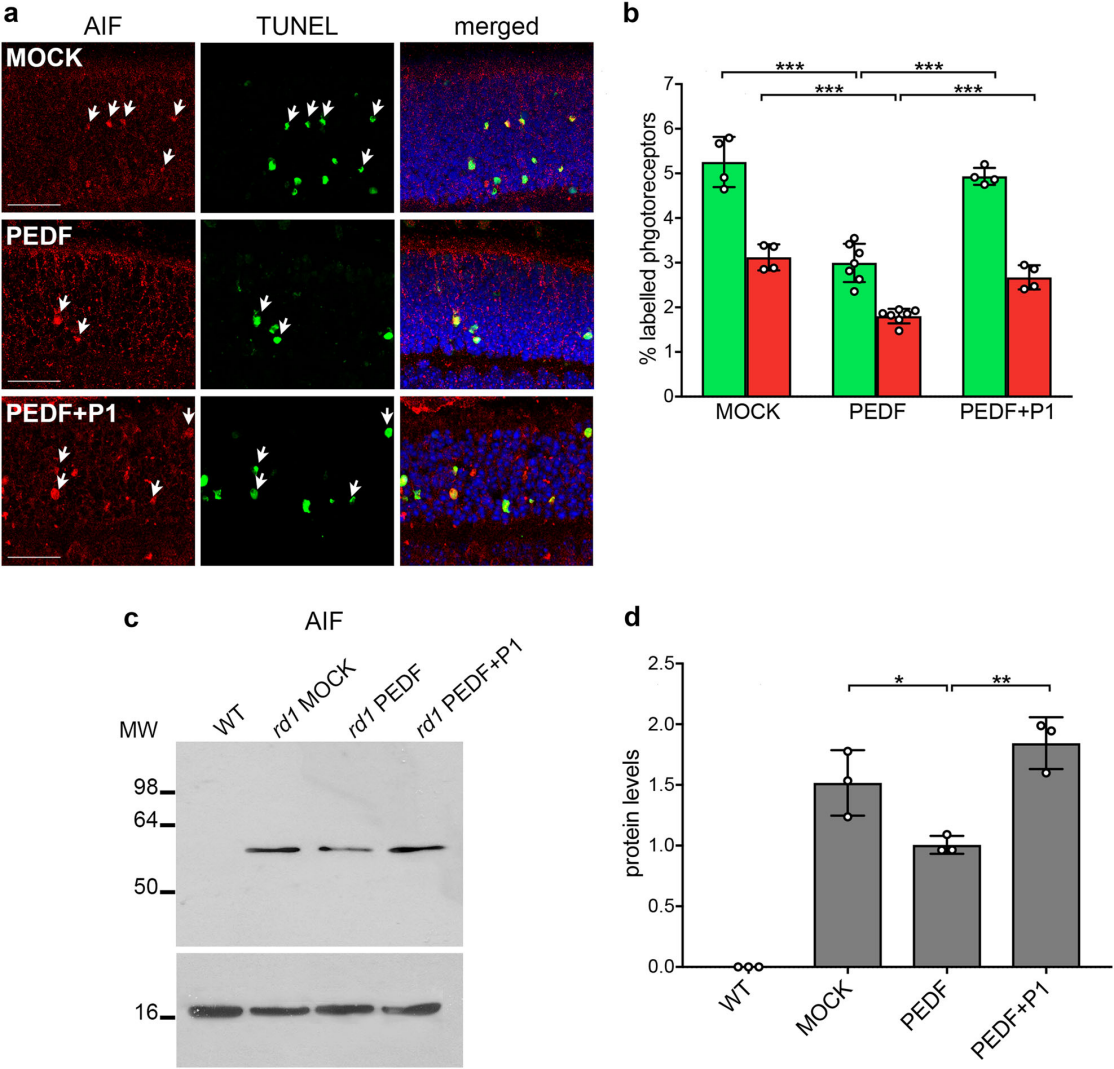
Effects of PEDF on AIF. **a** Immunofluorescence of AIF (red) and TUNEL^+^ staining of dying cells (green) in *rd1* mutant retinas treated with vehicle (MOCK) or PEDF or PEDF and 10 molar excess of P1 peptide (PEDF + P1). A decrease of double-labeled cells (arrows and yellow in the merged image) was observed after treatment with PEDF but not with PEDF + P1. Nuclei are stained in blue with DAPI. Only the outer nuclear layer containing photoreceptor nuclei is shown. Scale bars: 25 μm. **b** Histogram representing the percentage (±SD) of photoreceptors showing nuclear localization of AIF (red) or labeling with TUNEL (green). (*N* = 4–7; ***P ≤ 0.001). **c** Immunoblotting of AIF in nuclear-enriched protein extracts from wild-type (WT) and *rd1* mutant mouse retinas after treatment with either vehicle (MOCK) or PEDF or PEDF and P1 blocking peptide (PEDF + P1). The immunoblot was normalized with anti-H3 histone antibodies (lower panel). MW molecular weight markers are shown in kDa. **d** Quantification by ImageJ of three immunoblots from biological replicates as the one shown in panel (**c**) (±SD; *N* = 3; **P ≤ 0.01; *P ≤ 0.05)

Calpains are proteases that cleave mitochondrial AIF, a cell death executioner exiting from the mitochondria through a pore formed by BAX and translocating to the nucleus^39–41^. Here we performed in situ immunofluorescence staining and immunoblotting of nuclear extracts to evaluate AIF in *rd1* mutant retinas treated with PEDF. The data showed that PEDF decreased AIF nuclear levels implying that it restrained AIF nuclear translocation in the *rd1* murine model of retinal dystrophy (Figs. 4a–d and Supplemental Figures S5 d). The effects of PEDF on nuclear localization of AIF paralleled those on cell death evaluated by TdT-mediated dUTP terminal nick-end labeling (TUNEL) staining (Fig. 4b). The lack of reduction of AIF nuclear localization when PEDF was co-injected with the P1 peptide inferred that this effect was mediated by the binding to PEDF-R (Figs. 4a–d). Altogether these data defined that PEDF neuroprotection acted on the cell death pathway activated by calcium influx and mediated by AIF activation in photoreceptor cells.

### Retinoprotective domain of 17-amino acids signals like PEDF

**Fig. 5:**
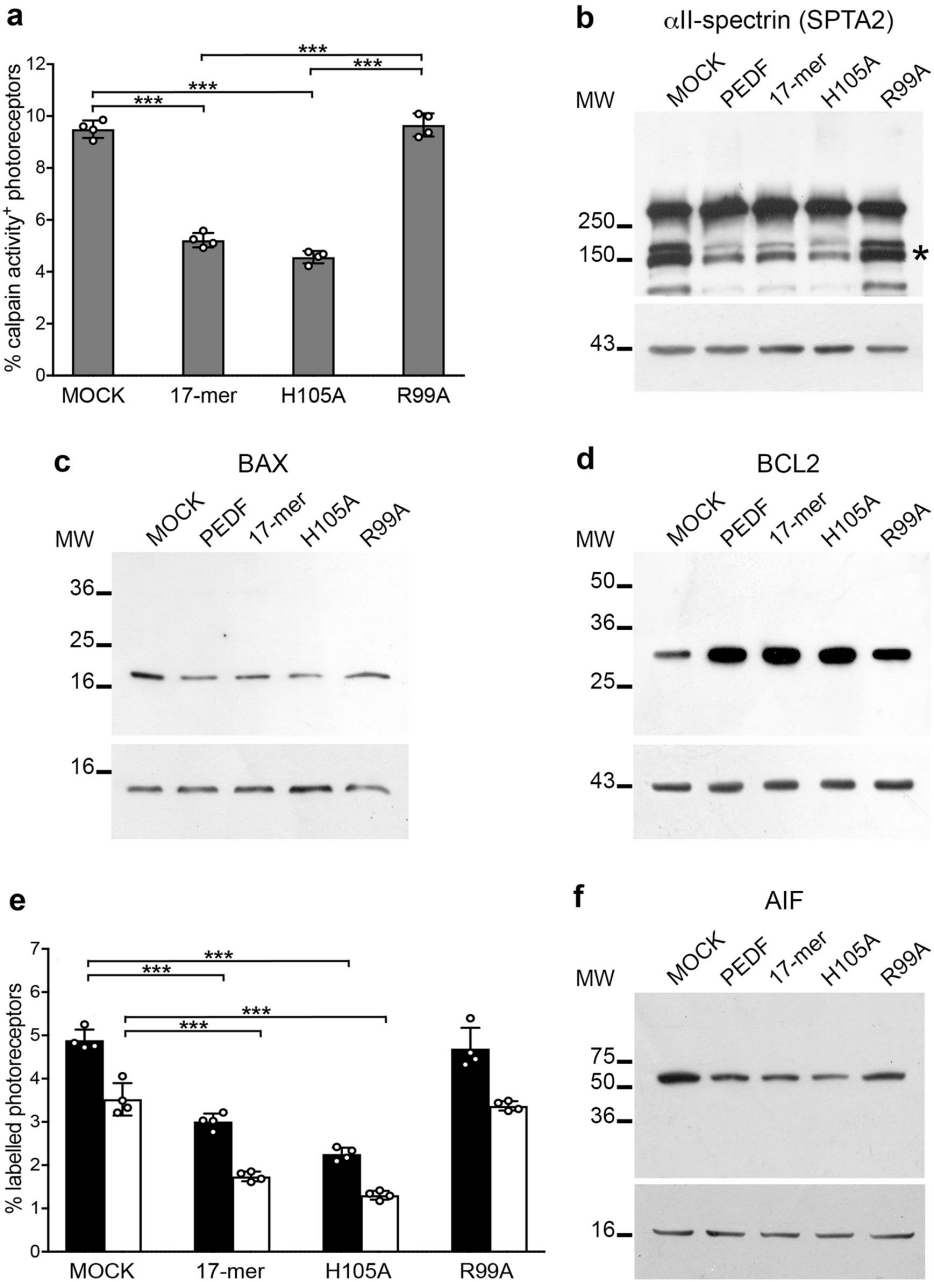
Neuroprotective activity of 17-mer peptides. Markers of the cell death pathways were analyzed in *rd1* mutant retinas after exposure to vehicle (MOCK) or 17-mer or 17-mer[H105A] (H105A) or 17-mer[R99A] (R99A). **a** Histogram representing the percentage of photoreceptors labeled by the fluorescent calpain activity assay (*N* = 4; ± SD; ***P ≤ 0.001). **b** αII-spectrin was analyzed by immunoblot in total protein extracts from mouse retinas. The 145–150 kDa fragments resulting from calpain cleavage are marked by an asterisk. The immunoblot was normalized using anti-actin antibodies (lower panel) (*N* = 3). **c** Immunoblotting of BAX protein in mitochondria enriched protein extracts. The immunoblot was normalized with anti-cytochrome *c* antibodies (lower panel) (*N* = 3). **d** Total protein extracts were analyzed by immunoblot with an anti-BCL2 antibody. The immunoblot was normalized using anti-actin antibodies (lower panel) (*N* = 3). **e** Histogram representing the percentage of photoreceptors labeled with TUNEL (black) or by nuclear localization of AIF (white) (*N* = 4; ± SD; ***P ≤ 0.001). **f** Immunoblotting of AIF protein in nuclear-enriched protein extracts. The immunoblot was normalized with anti-H3 histone antibodies (lower panel) (*N* = 3)

We previously mapped the retinoprotective domain of PEDF to 17-amino acids spanning between residue positions 98–114 of the human polypeptide sequence, here called 17-mer^11^. We hypothesized that the 17-mer domain mediated the effects of PEDF on the cell death effectors activated by increased calcium. To address this question, we intravitreally injected the 17-mer peptide or the altered 17-mer[R99A], with no PEDF-R binding affinity and neuroprotective activity, or the 17-mer[H105A], with an enhanced PEDF-R binding affinity and neuroprotective activity^11^. We found that peptides 17-mer and 17-mer[H105A] attenuated activation of calpains, BAX and AIF, whereas they increased BCL2 levels (Figs. 5a–f), similarly to what was observed with PEDF. In contrast, the 17-mer[R99A] peptide did not block the cell death pathways.

These results showed that the activity of the PEDF neurotrophic domain interfered with activation of calpains and, in turn, its downstream cell death effectors, and implied that interaction of amino acid positions 98–114 of PEDF with its receptor PEDF-R were sufficient for neuroprotection.

## Discussion

In this study, we report that PEDF challenges a cell death pathway in photoreceptor cells that is activated by increased intracellular Ca^2+^ in the degenerating rod photoreceptors bearing a loss-of-function mutation in the *Pde6b* gene. Our data demonstrate that this mechanism of neuroprotection is specifically mediated via the PEDF–PEDF-R interaction. In fact, the effect was abolished by the blocking PEDF-R peptide (PEDF-R ligand binding domain, P1 peptide). We also showed that the 17-mer and 17-mer[H105A] domains of PEDF, which bind PEDF-R, recapitulate the survival effect on degenerating photoreceptor cells like PEDF. Together with the absence of any survival effect by 17-mer[R99A], lacking binding affinity for PEDF-R, these observations further demonstrate that the PEDF neuroprotection activity on *rd1* photoreceptors is mediated by direct and specific interactions with PEDF-R.

**Fig. 6:**
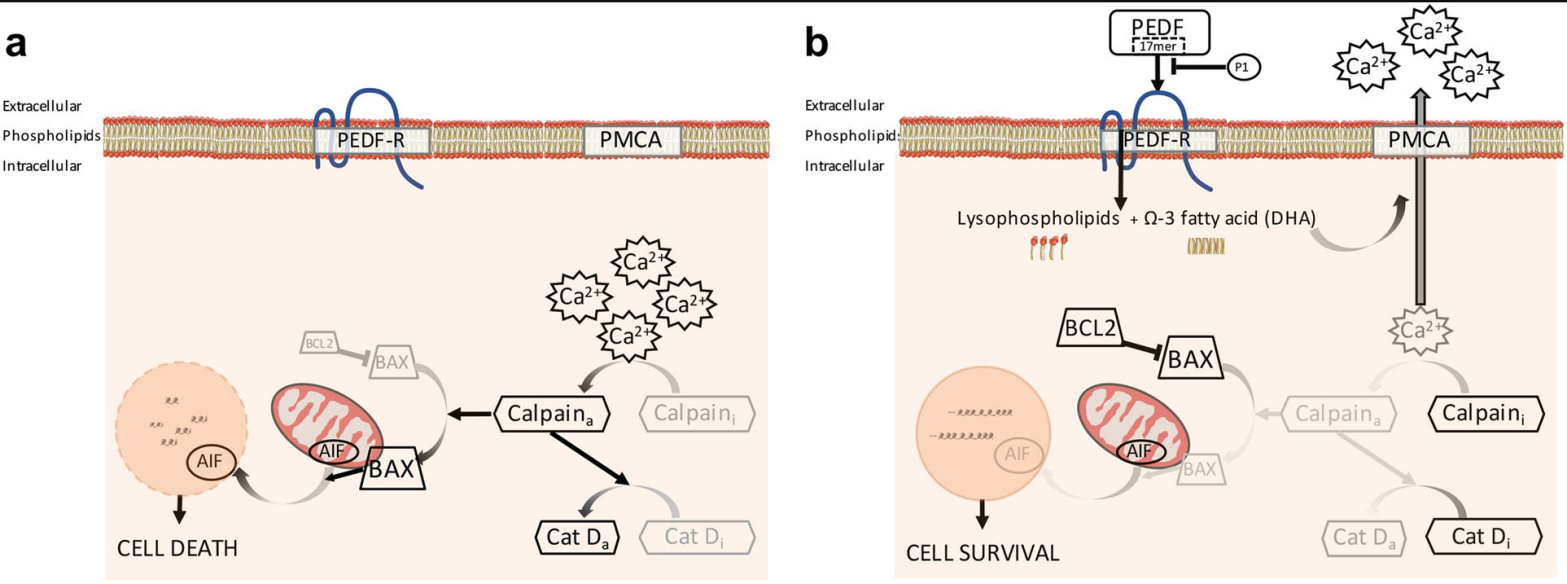
Scheme on the mechanism mediating PEDF promotion of photoreceptor survival. **a** Cell death pathway in *rd1* photoreceptors is associated with high [Ca^2+^]_i_, activation of calpain (Calpain_a_), Cathepsin D (Cat D_a_) and BAX, as well as of translocation of AIF from the mitochondria to the nucleus. **b** PEDF, or the 17-mer peptide, binds to PEDF-R and stimulates its PLA2 activity to release DHA from phospholipids (P1 peptide can interfere with the binding of PEDF to PEDF-R). DHA decreases [Ca^2+^]_i_ through PMCA activity (Ca^2+^ extrusion). Decreased [Ca^2+^]_i_ is associated with inactivation of calpain (Calpain_i_) and Cathepsin D (Cat D_i_), blockage of mitochondrial translocation of BAX and nuclear translocation of AIF, as well as increase in BCL2 levels, all of which contribute to cell survival in the *rd1* mouse photoreceptors

The binding of PEDF, specifically the domain containing the 17-mer, to PEDF-R on the cells surface activates intracellular events leading to cell survival (Fig. 6). The findings demonstrate that PEDF acts on survival via a calcium-related pathway. In fact, PEDF decreases the elevated levels of intracellular calcium, as well as the high calpain activity in *rd1* mutant photoreceptors. This effect is similar to the PEDF-mediated decreases of [Ca^2+^]_i_ in cerebellar granule cells exposed to glutamate toxicity, which have been associated with cell survival^47^. Interestingly, our data with inhibitors of calcium pumps shed light on the mechanisms by which PEDF diminishes intracellular calcium, by implying that PEDF favors calcium efflux, via PMCA, at the level of the plasma membrane and reduces cytoplasmic calcium below toxic levels. Recent reports demonstrated that PEDF–PEDF-R interactions stimulate the specific release of the omega-3 fatty acid docosahexaenoic acid (DHA) from phospholipids by the phospholipase A2 activity of PEDF-R^31,48^. It is worth noting that DHA can prevent Ca^2+^ overload by favoring PMCA function for Ca^2+^ efflux and by interfering L-type Ca^2+^ channels for Ca^2+^ entrance, shown both in cardiomyocytes^49,50^. On the one hand, we conclude that upon binding to the receptor, PEDF enhances its PLA2 activity to liberate DHA that targets PCMA favoring calcium efflux. At this time, we cannot exclude the effects on the L-type Ca^2+^ channel of DHA generated by the PEDF–PEDF-R interaction, which would be in agreement with our previous observations that blocking of this type of channels reduces photoreceptor cell death in the *rd1* mutant retina^37^. On the other hand, the effects of PEDF/PEDF-R on Ca^2+^ may also be mediated by the increase of BCL2, given that BCL2 modulates fluxes of Ca^2+^ in other systems^51^. Altogether, these observations suggest an effect of PEDF on intracellular targets preventing Ca^2+^ overload in *rd1* mutant photoreceptor cells.

As far as we know, this is the first time that a PEDF-mediated decrease in calpain activity is demonstrated. The neuroprotective effects of PEDF targeting intracellular calcium correlate with attenuation of the enzymatic activities of calpains and of cathepsin D, which together are involved in triggering BAX and AIF cell death mechanisms^2,37^. The effect on calpains could again be mediated by DHA as this fatty acid, a mediator of PEDF signaling^52^, could interfere in vitro with calpain activation in cells stressed by Ca^2+^ influx^53^. PEDF also weakens the cell death pathway involving BAX and AIF. Our data agree with the report by Murakami et al, in which PEDF administration in the retina of Royal College of Surgeon rats upregulates the *Bcl2* gene and blocks AIF nuclear translocation in photoreceptors^12^. Our previous report on the protection of PEDF on DKO *rd8* (Ccl2^-/-^/Cx3cr1^-/-^ on C57BL/6 N [Crb1^rd8^]) mice, a model for progressive, focal retinal degeneration, agrees with data in the present study by showing lower levels of BAX protein, higher levels of BCL2 protein, decrease in cell death and attenuation of focal retinal lesions in the PEDF-treated retina^20^.

In summary, our data highlight the actions of PEDF and PEDF-based peptides as neuroprotective factors for photoreceptor cells at early steps during the cell death cascade thus affecting several molecules correlated to photoreceptor cell degeneration. The conclusions enhance our understanding of the molecular mechanism underlying the effects of a neurotrophic factor targeting common cell death mechanisms, which is an attractive strategy for treating more than one form of the many types of retinal degenerations.

## Materials and methods

### Animal care and treatments

All procedures on mice were conducted at CSSI (Centro Servizi Stabulario Interdipartimentale), approved by the Ethical Committee of University of Modena and Reggio Emilia and by the Italian Ministero della Salute (346/2015-PR) and were in accordance with the ARVO Statement for the Use of Animals in Ophthalmic and Vision Research. C3H/HeN (*rd1*) mice were purchased from Envigo Italy (Udine, IT). Mice were maintained in 12-h light/dark cycles and had free access to food and water. For intravitreal administration, mice were anesthetized with an intraperitoneal injection of 250 mg/kg body weight of avertin (1.25% (w/v) 2,2,2-tribromoethanol and 2.5% (v/v) 2-methyl-2-butanol; Sigma, Milan, IT). Subsequently, eyes were intravitreously injected via a trans-scleral trans-choroidal approach with 0.5 μl of PEDF recombinant protein (6 pmol) or of PEDF recombinant protein (6 pmol) with 100 μM CLX 3A1 (final intravitreal concentration; AnaSpec) or of 17-mer (6 pmol) or of 17-mer[R99A] (6 pmol) or of 17-mer[H105A] (2 pmol) peptides or of PEDF together with 10-fold molar excess of P1 peptide (a peptide from PEDF-R encompassing the PEDF binding domain)^11^. Control eyes received vehicle only (mock injected). Mice were sacrificed 16 h post-injection.

### Cell culture and treatments

661W cells, immortalized photoreceptor precursors^54^, were generously provided by Dr. Muayyad Al-Ubaidi (University of Oklahoma) and cultured in high glucose (4.5 mg/ml) Dulbecco’s modified Eagle's medium (DMEM) supplemented with 10% fetal bovine serum (FBS), 2 mM glutamine, 100 U/ml penicillin and 100 µg/ml streptomycin purchased from Invitrogen (ThermoFisher Scientific). In total, 20,000 cells were seeded onto glass cover-slips coated with poly-d-lysine. The following day cells were treated for 2 h with medium containing 10 nM PEDF or an equal volume of phosphate-buffered saline (PBS) as control and then stressed with 500 μM zaprinast (Sigma) or 5 μM A23187 (Sigma) for 16 h in the absence or presence of PEDF. When calcium pump blockers were used, 100 μM BZ (Tocris) or 10 μM CLX (AnaSpec) or 200 nM TG (Sigma) were added. Cells were fixed with 2% paraformaldehyde (PFA) for 10 min at room temperature.

Retinal neurospheres were generated from *rd1* mutant eyes and differentiated into rod-like cells as previously published^37^. Cells were exposed to 10 nM PEDF or 10 nM PEDF with 10 μM CLX or an equal volume of PBS as control at 10 days of culture in differentiation medium (1 day before the peak of cell death^37^). Eighteen hours after treatment, cells were fixed with 2% PFA for 10 min.

Either 661W cells or primary rod-like cells underwent TUNEL assay or Fluo-4 AM staining, as described below. Nuclei were stained with 0.0005% 4′,6-diamidino-2-phenylindole (DAPI; Molecular Probes). Slides were mounted with Mowiol 4–88 (Sigma), images were taken using a Zeiss Axio Imager A2 microscope and TUNEL^+^ and DAPI-stained nuclei were counted to calculate percentage of cell death.

### Cytofluorimetric analysis of calcium

Intracellular calcium levels were determined with the intracellular calcium probe Fluo-4 AM (Life Technologies), as previously published^55^. Briefly, retinas were harvested and incubated in 19 U/ml papain for 30 min and, after 33-fold dilution with DMEM containing 10 U/ml DNAse, retina cells were dissociated by trituration. After three washes with PBS, cells were incubated with 0.5 μM Fluo-4 AM at 37 °C for 30 min in Ca^2+^-free medium. Fluorescence was measured with a Coulter Epics XL-MCL flow cytometer (Beckman Coulter) at an excitation wavelength of 488 nm and Fluo-4 AM median fluorescence intensity (MFI) was calculated. Photoreceptor cells were characterized by co-staining with anti-Rhodopsin antibody 1D4 (1:1000, Sigma) and anti-Recoverin (1:500, Millipore) as described before^56^ and plotted over the forward scatter to define the gating strategy for the following intracellular calcium analysis (Supplemental figure S1 a and b). Fluo-4 AM signal was measured in at least four different *rd1* retinas treated with PEDF and the percentages of cells with high fluorescence were compared with contralateral *rd1* retinas either mock-treated or treated with PEDF and 10-fold molar excess of P1 peptide. The ratios are presented as means ± SD of 4–8 biological replicates.

Cytofluorimetric analysis of calcium in 661W cells was performed as in Wyse-Jackson et al.^57^. Briefly, cells were stained with 1 μM Fluo-4 AM at 37 °C for 30 min in Ca^2+^-free medium Hank’s Balanced Salt solution (HBSS, Thermofisher) in the dark. After incubation, cells were washed twice with PBS. Cell were detached by treatment with Accutase^TM^ (Millipore) and resuspended in 10% HBSS, 1 mM CaCl_2_, 1 mM MgCl_2_, 1% FBS. Finally, cells were washed in annexin binding buffer and incubated 10 min with annexin V conjugated with Pacific Blue (Thermofisher) and the TO-PRO™3 Ready Flow™ Reagent (Thermofisher) was added just before the acquisition according to standard procedures. Cells were acquired by using an Attune NxT Acoustic flow cytometer (Thermofisher) equipped with four lasers (405 nm, 488 nm, 561 nm, 634 nm). Living cells (TO-PRO-Annexin V^−^) were gated (Supplemental figure S1 e), and Fluo-4 AM MFI was calculated on such cells.

### Calpain activity assay

Cryosections from unfixed retinas of at least three mice were incubated for 15 min in calpain reaction buffer (CRB: 25 mM HEPES-KOH pH 7.2, 65 mM KCl, 2 mM MgCl_2_, 1.5 mM CaCl_2_, 2 mM DTT), as previously described^9^, and then exposed for 1 h at 37 °C to the fluorescent calpain substrate CMAC, t-BOC-Leu-Met (A6520, Invitrogen) at a final concentration of 2 µM. Slides were washed twice for 10 min each in CRB, mounted with mowiol 4–88 (Sigma) and analyzed at a Zeiss Axio Imager A2 microscope using the filter excitation/emission 365/420. Labeled cells were counted in the outer nuclear layer (ONL) containing photoreceptors in at least three entire sections passing through the optic nerve from three independent experiments.

### DNA nick-end labeling by TUNEL and immunofluorescence

Eyes were oriented, fixed in Davidson’s fixative (8% Formaldehyde, 31.5% Ethanol, 2 M Acetic Acid), embedded in paraffin and sectioned as previously described^37^. Apoptotic nuclei were detected by TUNEL kit (fluorescein; Roche) according to the producer’s protocols. Primary antibodies were used as follows: anti-AIF (1:100; Sigma), anti-phosphorylated PERK (1:100, Cell Signaling), anti-RHO (1D4 1:250; Sigma), anti-BAX 6A7 (1:200, BD Biosciences), anti-BCL2 (1:100, Cell Signaling) and anti-calpastatin (1:100, Cell Signaling). Secondary antibodies were Alexa Fluor^®^ 568 anti-mouse and anti-rabbit antibodies (1:1000, Molecular Probes). Slides were mounted with mowiol 4–88 (Sigma) and analyzed at a Zeiss Axio Imager A2 microscope. Quantification of dying cells was performed by counting all TUNEL labeled cells in the photoreceptor cell layer passing through the optic nerve in at least three sections from different animals. The number of photoreceptors was counted by staining of nuclei with DAPI and was used to calculate percentage of dying cells.

### Retinal protein extracts and western blotting analysis

Retinas were lysed as previously described to obtain nuclear-enriched proteins and mitochondrial-enriched proteins^2^. The purity of enriched lysates was checked by immunoblotting using a nuclear marker (anti-Histone H3 1:3000; Bethyl Laboratories), a mitochondrial marker (anti-cytochrome c, 1:2000, BD Biosciences) or a cytosol marker (anti-pan-actin, 1:3000, Millipore).

Equivalent amounts of protein extracts (20 μg) were resolved using sodium dodecyl sulfate–polyacrylamide gel electrophoresis and immunoblottings were performed following standard procedures. The antibodies used for western blotting were: anti-αII-spectrin (1:2000, Enzo Life), anti-AIF (1:1000, Calbiochem), anti-BAX 6A7 (1:2000, BD Biosciences), anti-BCL2 (1:2000, Cell Signaling), anti-calpastatin (1:1000, Cell Signaling), anti-cathepsin D (C-20 1:1000, Santa Cruz Biotechnology). Quantification was performed by densitometry analysis of scanned images with the ImageJ software, corrected by background and plotted as protein/normalizing protein. Data are presented as means ± SD of three blots. Each blot analyzed proteins derived from four retinas pooled together and three independent pools from three different litters were used as biological replicates.

### Statistical analysis

Cell counts and densitometry analyses are shown as mean ± SD. Student’s *t*-test analysis was performed to compare data derived from at least three different mock-treated mutant retinas or retinas co-treated with PEDF and 10-fold molar excess of P1 peptide to at least three different contralateral PEDF-treated mutant retinas.

## Electronic supplementary material

Supplementary figures
