# Supplementary material for: Pigment epithelium-derived factor hinders photoreceptor cell death by reducing intracellular calcium in the degenerating retina
Source: Cell Death Dis. 2018 May 11;9(5):560. doi: 10.1038/s41419-018-0613-y (PMC5948223; doi:10.1038/s41419-018-0613-y)
Supplement: Supplementary file 1 — Supplementary figures [file 41419_2018_613_MOESM1_ESM.pdf]

Figure S1

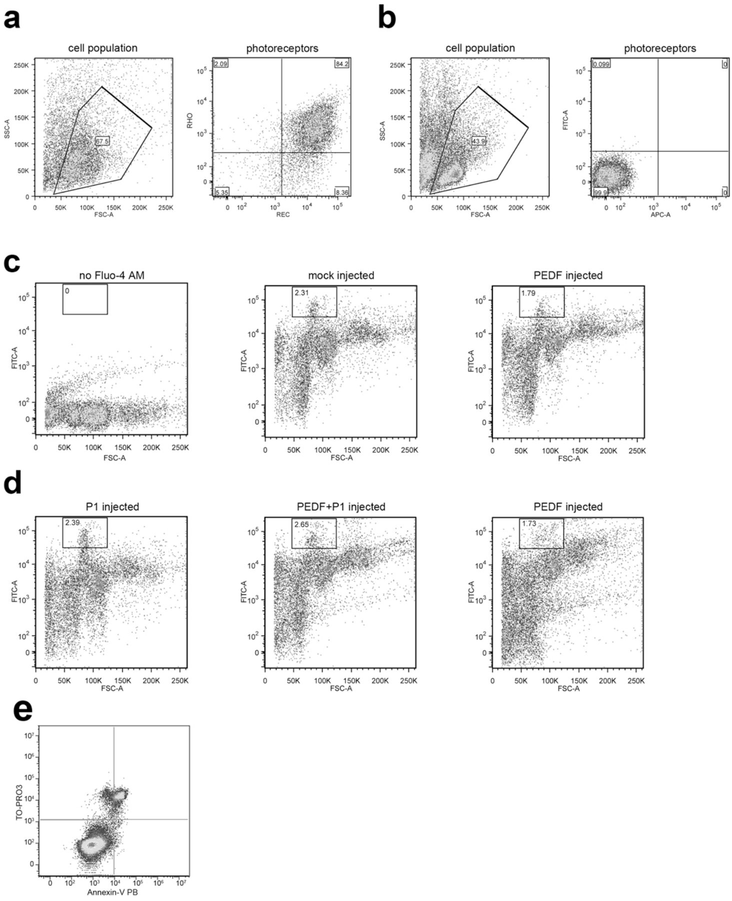

Figure S1. Flow cytofluorimetric analyses of calcium. (**a**) Flow cytometric characterization of photoreceptor cells dissociated from a PN12 *rd1* mutant retina. Shown are the analyses of the cell population and of the rod photoreceptor cells labeled with the anti-Rho antibody (Y axis, RHO) and anti-recoverin (X axis, REC). This photoreceptor population was gated for studies shown in figure 1 a and b. (**b**) Flow cytometry unstained controls of the cell population and of the secondary antibodies (FITC = fluorescein; APC = Allophycocyanin) used for analyses shown in panel a. (**c**) Flow cytofluorimetry outcomes of calcium labeling with Fluo-4 AM (fluorescence intensity on the Y axis) in *rd1* retinas at PN12 either mock injected or injected with PEDF. Cell with high level of Ca^{2+} were measured by gating as shown in the figure. The percentage values retrieved by the gating were used to calculate ratios shown in figure 1b. The background fluorescence in samples not treated with Fluo-4 AM (no Fluo-4 AM) is shown in the panel at the left-hand side. (**d**) Flow cytofluorimetry outcomes of calcium labeling with Fluo-4 AM (fluorescence intensity on the Y axis) in *rd1* retinas at PN12 either injected with P1 alone or co-injected with PEDF and P1 blocking peptide or injected with PEDF. The gates applied measured cells with high level of Ca^{2+} and these values were used to calculate ratios shown in figure 1b. (**e**) Characterization of the cell population used for cytofluorimetric analysis of 661W cells. Only non-apoptotic cells, negative to TO-PRO3 and annexin-V staining (lower left quadrant in the figure), were analyzed for calcium content shown in figure 1c.

Figure S2

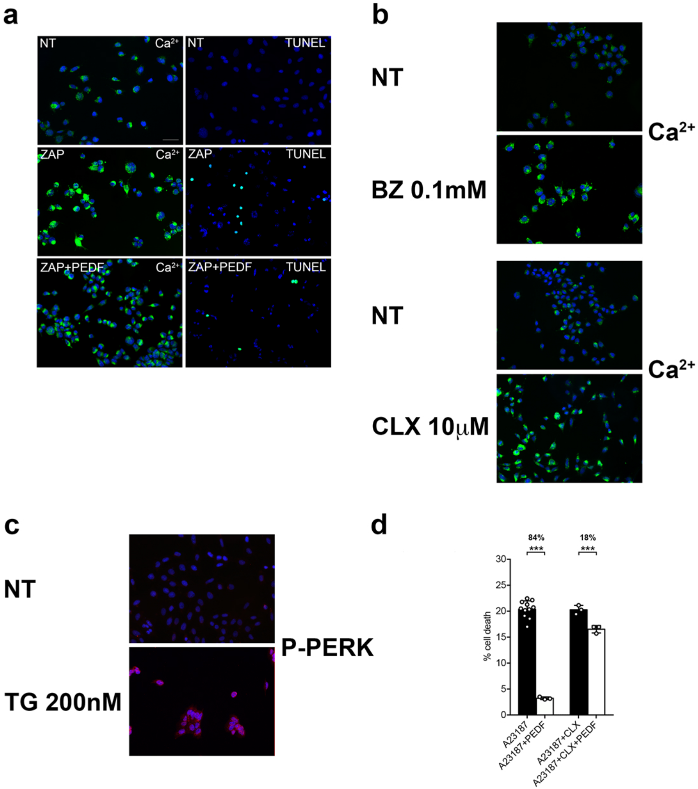

Figure S2. Effects of calcium pump blockers in 661W photoreceptor cells. (**a**) 661W cells stressed with 500 μM zaprinast (ZAP) showed increased intracellular Ca^{2+} by staining with Fluo-4 AM (green in left-hand side panels) and cell death (TUNEL, green in right-hand side panels) when compared to not treated cells (NT). PEDF (ZAP+PEDF) reduced intracellular Ca^{2+} (left-hand side panels) and cell death (right-hand side panels). (**b**) 661W cells were treated for 16 hours with either 100 μM 3',4'-dichlorobenzamyl (BZ) or 10 μM caloxin (CLX). The blocking effects of BZ and CLX on the pumps was demonstrated by increased intracellular Ca^{2+} visualized by Fluo-4 AM staining (green signal). (**c**) The effect of 200 nM thapsigargin (TG) was assessed by analysis of activation of the ER-stress sensor PERK that was found phosphorylated (red signal). (**d**) 611W cells were treated for 16 hours with 5 μM A23187, an ionophore that allows calcium influx. Influx of Ca^{2+} induced cell death in about 20% of the cells. Exposure to 10 nM PEDF reduced cell death by 84%. When cells were co-treated with the calcium pump blocker caloxin (CLX, 10 μM), PEDF neuroprotective effects were almost lost with a remaining neuroprotection of 18%. Data are shown as means ±SD (N=3; *** P≤0.001).

Figure S3

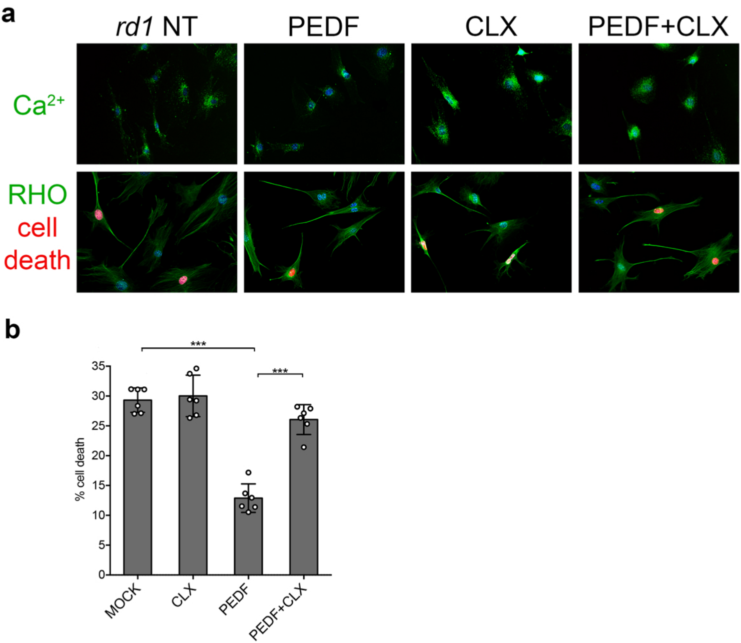

Figure S3. PEDF protects primary *rd1* mutant rod-like cells against cell death by acting on PMCA. (**a**) Retinal neurospheres were generated from *rd1* mutant ciliary epithelium and allowed to differentiate into rod-like cells for 11 days. At day 10 of differentiation, cells were exposed to 10 nM PEDF or to PBS as control (*rd1* NT) or to 10 μM caloxin (CLX) or to 10 nM PEDF and 10 μM caloxin (PEDF+CLX). Eighteen hours later cells were fixed and analyzed for intracellular Ca^{2+} with Fluo-4 AM (green, upper panels) and for cell death (red, lower panels) and rhodopsin expression (RHO green, lower panels). PEDF decreased intracellular Ca^{2+} and cell death. (**b**) PEDF (10 nM) recovered cell death by 42% of primary *rd1* mutant rod-like cells. PEDF neuroprotection was lost when cells were exposed to 10 μM caloxin (CLX). Data are shown as means ±SD (N=6; *** P≤0.001).

Figure S4

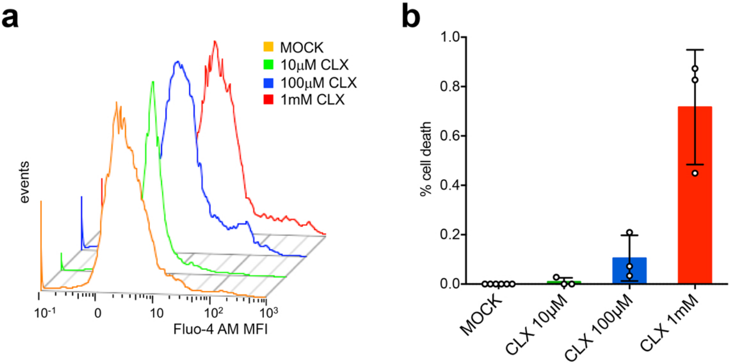

Figure S4. Effects of different caloxin concentrations *in vivo* in degenerating retinas. Eyes were intravitreally injected with 10 or 100 or 1000 μM caloxin at PN11 and analyzed at PN12. (**a**) The levels of calcium (medium fluorescence intensity, MFI) was analyzed by Fluo-4 AM in photoreceptor cells (events), characterized as described in supplemental figure S1. Significant increase of calcium was detected starting from treatments with 100 μM of caloxin, suggesting that this concentration was sufficient to affect PMCA pumps in photoreceptor cells *in vivo*. (**b**) Photoreceptor cell death was evaluated in sections of treated retinas and the concentration of 100 μM of caloxin induced less than 1% of cell death in photoreceptors.

Figure S5

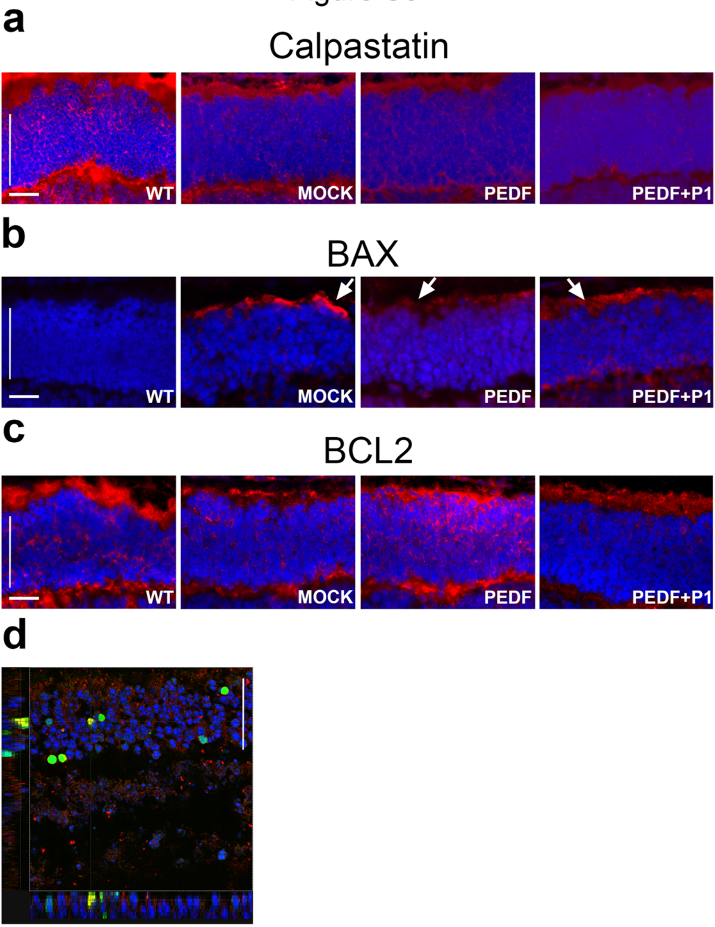

Figure S5. Effects of PEDF on Calpastatin, BCL2 and BAX. (**a**) Calpastatin was evaluated by immunofluorescence (red). Decreased Calpastatin was detected in *rd1* mock treated retina (MOCK) compared to wild type retina (WT). Nuclei were stained with DAPI (blue). No change was detected after treatment with PEDF (PEDF) or PEDF and P1 blocking peptide (PEDF+P1). (**b**) Activation of BAX was detected with the anti-activated-BAX specific antibody (red) at the inner segment (containing cytoplasm, mitochondria and ER of photoreceptor cells) of *rd1* treated mutant retinas (MOCK; arrow). No staining with the antibody for activated BAX was detectable in wild type retina (WT) at P12. Fluorescent staining was reduced after treatment with PEDF (arrow) but not after treatment with PEDF and P1 blocking peptide (PEDF+P1; arrow). (**c**) BCL2 was evaluated by immunofluorescence (red). Nuclei were stained with DAPI (blue). Decreased BCL2 was detected in *rd1* mock treated retina (MOCK) compared to wild type retina (WT). Recovery of BCL2 was detectable after treatment with PEDF (PEDF) but not in retinas treated with PEDF and P1 blocking peptide (PEDF+P1). Micrographs in panels a, b and c show the outer nuclear layer containing photoreceptor cells (white bars). Scale bar in panels a, b and c: 20μm. (**d**) *rd1* mutant retina section stained with antibodies anti-AIF (red), TUNEL assay (green) and DAPI (blue). Confocal xy-planes and the z-stack are shown. Red, green and blue fluorochromes label the selected dying cell, as shown at the right-hand side. Fluorescence of the three channels along the horizontal line passing through the same co-labelled cell is shown below the micrograph. The outer nuclear layer, containing photoreceptor nuclei, is indicated by a white bar.
